# Point-of-care biomarkers or laboratory-based tests to reduce antibiotic prescribing for COPD exacerbations: a literature review and meta-analysis in primary care

**DOI:** 10.3399/BJGP.2025.0593

**Published:** 2026-07-14

**Authors:** Mathieu Frénois, Roxane Fovet, David Wyts, Claire Collins, Matthieu Calafiore, Christophe Berkhout

**Affiliations:** 1 District of General Practice, Department of Medicine, University of Lille, Lille, France; 2 Irish College of General Practitioners, Dublin, Ireland; 3 Department of Public Health and Primary Care (GE39), Faculty of Medicine and Health Sciences, Ghent University, Ghent, Belgium; 4 senior researcher, Metrics, Department of Medicine, University of Lille, Lille, France; 5 associate professor, Department of Family Medicine, Faculty of Medicine and Health Sciences, McGill University, Montréal, Canada; 6 and director, District of General Practice, Department of Medicine, University of Lille, Lille, France; 7 and senior researcher, ELIZA research group, Department of Family Medicine and Population Health, University of Antwerp, Antwerp, Belgium

**Keywords:** antibiotic, biomarker, COPD, C-reactive protein, point-of-care testing, procalcitonin

## Abstract

**Background:**

Acute exacerbations of chronic obstructive pulmonary disease (AECOPD) are frequently treated with antibiotics in primary care, leading to antibiotic resistance and adverse effects. Assessing biomarkers might assist in reducing unnecessary antibiotic prescribing when used alongside clinical assessment.

**Aim:**

To evaluate the benefit of reviewing biomarkers in point-of-care testing (POCT) within practices and through laboratory blood tests in efforts to decrease antibiotic prescribing for AECOPD.

**Design and setting:**

Systematic review of randomised controlled trials (RCTs) and non-randomised studies conducted in primary care from January 2022 to November 2025 assessing POCTs for chronic obstructive pulmonary disease (COPD).

**Method:**

The primary outcome was the rate of antibiotic prescribing or use, and secondary outcomes were biomarker thresholds for prescribing antibiotics and patient harm. Searches were conducted in four medical databases, six registers, and COPD academic societies. Data were extracted and assessed for quality and bias. A meta-analysis was performed whenever possible.

**Results:**

A total of seven studies were included: one RCT and four non-randomised studies for C-reactive protein (CRP) POCT; and two RCTs on procalcitonin (PCT) in laboratories. The meta-analysis of CRP POCT indicated a statistically significant reduction in antibiotic prescribing (odds ratio 0.41, 95% confidence interval = 0.32 to 0.53; *I*² = 36%; low certainty of evidence). No meta-analysis for PCT was feasible.

**Conclusion:**

CRP POCT reduced antibiotic prescribing for AECOPD in primary care settings when used alongside clinical assessment. There were insufficient data to draw conclusions about the PCT biomarker.

**How it fits in**

Acute exacerbations of chronic obstructive pulmonary disease (AECOPD) represent a common primary care situation in which the choice of whether to prescribe an antibiotic is difficult. In addition to clinical examination, the use of biomarkers such as C-reactive protein (CRP) in point-of-care testing (POCT) or procalcitonin (PCT) in laboratories could help in the decision making on whether to prescribe an antibiotic. Rapid CRP POCTs reduced antibiotic prescribing without increasing respiratory risk for AECOPD in primary care, but the level of certainty was low. There are insufficient data to conclude on the usefulness of PCT in laboratories in reducing antibiotic prescribing for AECOPD in primary care.

## Introduction

GPs diagnose about 80% of acute exacerbations of chronic obstructive pulmonary disease (AECOPD) in primary care settings.^1^ GPs usually manage them with bronchodilators, corticosteroids, or antibiotics.^2^ Limiting unnecessary antibiotic prescribing in primary care is vital in reducing bacterial resistance to antibiotics at both societal^3,4^ and individual levels.^5^ Antibiotic resistance leads to ineffective treatments, increased risk of serious complications, such as bacterial infections and death, and increased healthcare costs.^6,7^ Antibiotics have side effects and drug interactions that may harm patients. However, patient safety must also be carefully considered by the GP to reduce the risk of undertreatment of serious bacterial infections.

The decision on whether to prescribe antibiotics for an AECOPD is challenging. On one hand, it reassures both GPs and patients but, on the other hand, only bacterial AECOPD may benefit from antibiotics and the majority of AECOPD are not caused by bacteria (bacterial AECOPD account for 20%–50% in clinical samples)^8,9^ but rather by virus or environmental factors, such as air pollution. Clinical signs of bacterial AECOPD may not be easily identified by the GP and are controversial. Since 1987,^10^ Anthonisen criteria are often used with increased dyspnoea, increased sputum volume, and increased sputum purulence, even though they are not specific to bacterial infection as they may be caused by local inflammation.^11^ These signs are subjective and remain vague in identifying whether a patient could be treated without antibiotics.

In 2018, Vollenweider *et al* updated a Cochrane systematic review from 2012 with 19 randomised trials (*n* = 2663 participants) about the value of antibiotics for inpatients and outpatients with AECOPD.^12^ It reported inconsistent effects of antibiotics and called for measuring biomarkers through blood tests to identify who would benefit from antibiotics, while avoiding antibiotics for patients who are unlikely to experience benefits and for those who may experience antibiotic drawbacks. The US Food and Drug Administration and National Institutes of Health define a biomarker as a biological molecule found in blood, other body fluids, or tissues that is a sign of a normal or abnormal process, or of a condition or disease.^13^ Another systematic literature review^14^ and a trial^15^ have already explored many biomarkers but mainly in secondary care settings. Such biomarkers were either very specialised and not relevant to primary care, or participant recruitment spanned undifferentiable primary and secondary care, making them unusable for primary care patients.^14^ In 2022, Smedemark *et al*, in another Cochrane systematic review, explored the use of point-of-care tests (POCTs) in primary care for upper and lower respiratory tract infections alongside clinical examination and concluded a likely reduction of antibiotic prescribing due to C-reactive protein (CRP) POCTs, with moderate to high certainty of evidence.^16^ It emphasised the need for further analysis on specific subgroups such as immunocompromised patients, children, and older people. Studies show that, for the ranges useful in general practice, rapid CRP POCTs are sufficiently concordant with laboratory measures to guide management.^17^

To date, to the authors’ knowledge, no systematic review has considered biomarkers specifically for the AECOPD population in primary care settings to help reduce antibiotic prescribing.

The aim of this systematic review and meta-analysis was to assess the benefit of POCTs in GPs’ practices or blood tests in laboratories to reduce unnecessary antibiotic prescribing for AECOPD in primary care settings.

## Method

This systematic review of literature applied the Preferred Reporting Items for Systematic Reviews and Meta-Analyses 2020 statement^18^ and the *Cochrane Handbook for Systematic Reviews and Interventions*.^19^ A Cornell University diagram was used to ensure this work met systematic literature review criteria.^20^ The protocol of this review was registered on PROSPERO, an international prospective register of systemic reviews (registration number: CRD42024575414).

### Eligibility criteria

The primary outcome was the rate of antibiotic prescribing or use, and secondary outcomes were biomarker thresholds for prescribing antibiotics and patient harm.

To be eligible, studies had to have a primary outcome related to a POCT or a laboratory blood test to reduce unnecessary antibiotic prescribing for AECOPD in primary care settings. Included studies had to report the following criteria:

- proven diagnosis of COPD with spirometry (any GOLD stage) and/or diagnosis of COPD in primary care clinical records;^2^
- only primary care patients or separate analysis of primary care patients;
- patients presenting with an AECOPD with at least one of the Anthonisen criteria (increased dyspnoea, increased sputum volume, increased sputum purulence, and lasting a minimum of 24 hours and a maximum of 21 days);^10^ and
- blood test as POCT, or in a laboratory, to help decision making about antibiotic prescribing or use.

The authors excluded studies that reported:

- hospital patients (emergency or medical wards or intensive care unit); or
- patients requiring urgent hospital admission.

Randomised controlled trials (RCTs), cohort studies, retrospective, cross-sectional, before-after, and case–control studies were included. Systematic literature reviews, meta-analyses, qualitative studies, editorial letters, or comments were excluded.

### Information sources

A first review of literature was conducted from 1 January 2022 to 31 August 2023, with an updated search carried out from 1 September 2023 to 30 November 2025 in four medical databases: MEDLINE (1977–2023 then 2023–2025); the Cochrane Central Register of Controlled Trials (CENTRAL) (1948–2023 then 2023–2025); Embase (1975–2023 then 2023–2025); and the Web of Science (1900–2023 then 2023–2025), in the same months, respectively. The following registers were explored: ClinicalTrials.gov; Australia New Zealand Trauma Registry; EU Clinical Trials Register; Dutch Trial Register; UK International Standard Randomised Controlled Trial Number registry; and US Clinical Trials Transformation Initiative. The authors also explored COPD academic societies’ websites.

### Search strategy

A research equation by database was built in several steps:

1. Breaking down the research aim: *‘Assessment of the benefit of POCT in GPs’ practice or blood test in laboratories to reduce unnecessary antibiotic prescribing for AECOPD in primary care settings’* into three blocks. Each block resulted in one research equation with semantic specificities related to each database.
2. Breakdown resulted in Block 1: ‘COPD’; Block 2: ‘Antibiotics’; and Block 3: ‘Biomarkers’.
3. One final research equation per database merged the three blocks.

The export feature in the four databases was used by generating a .ris or .nbib file enabling an import in the ZOTERO tool (version 5.0.96.4 in February 2022 to version 7.0.30 in November 2025). Then, a ZOTERO group library was created to remove duplicates. The search was not restricted to any language.

### Selection and data collection process

Resulting records on ZOTERO after removing duplicates were then imported into the Rayyan platform (version 1.6.0), to select reports by title and abstract.^21^ Two independent reviewers, and occasionally three reviewers on specific points, blind to their allocations proceeded with report selection. The reviewers could choose three allocations: ‘yes’, ‘no’, and ‘maybe’ if researchers were unsure. The ‘maybe’ reports were then allocated either to ‘yes’ or ‘no’ after consensus between reviewers or discussion with the supervisor.

Included reports remained blinded during this process and were then imported back into ZOTERO.

Citations and bibliographical references at the end of included reports and excluded systematic reviews of literature and meta-analysis were also investigated with a tool called ‘citationchaser’ (version 0.0.4), offering two features ‘forward citation chaser’ and ‘backward citation chaser’ to ensure no relevant reports were missed.^22^

### Data items

All reports referring to inpatients were excluded. Reports referring to patients with lower respiratory tract infection (LRTI) were only included if a relevant AECOPD primary care subgroup was created and analysed. A single researcher extracted the relevant data for analysis purposes. Any primary or secondary outcome was considered, and any limitation or missing information was considered as a bias.

### Study risk of bias assessment

The risk of bias of the included reports was assessed by two independent investigators using the Cochrane tool, as explained in the *Cochrane Handbook for Systematic Reviews of Interventions.* ^19^ RobVis (version 0.3.0) was used to generate risk-of-bias assessment figures.^23^

The quality of reports was assessed using a standard tool to assess risk of bias according to the Grading of Recommendations Assessment, Development, and Evaluation (GRADE) framework.^24^

### Effect measures and synthesis method (meta-analysis)

The effect of biomarkers was reported as an odds ratio (OR) with a 95% confidence interval (CI). The adjusted OR within reports, when available, was input in Cochrane Review Manager (RevMan, version 5.3). When the OR was not available in reports, computation through RevMan with four values from reports was performed.

Unpublished measures were not sought. Generic inverse variance was used, adjusting for the direction of the effect (that is, increase or decrease). OR and 95% CI were computed using random effects in the context of an important difference in weight of the studies. Heterogeneity was computed using the *I*² statistic and the heterogeneity χ^2^. The result was presented as a forest plot.

### Reporting bias assessment

A RevMan funnel plot assessed potential missing results for the primary outcome.

#### Certainty of evidence

Certainty of evidence for the meta-analysis was aggregated from the individual level of evidence (GRADE) for each report included in the meta-analysis. Three sensitivity analyses were conducted to assess the robustness of the synthesised results: one with non-randomised studies only; one with the three best quality reports; and another giving a clear cut-off value for biomarker intervention.

## Results

### Study selection

**Figure 1.**
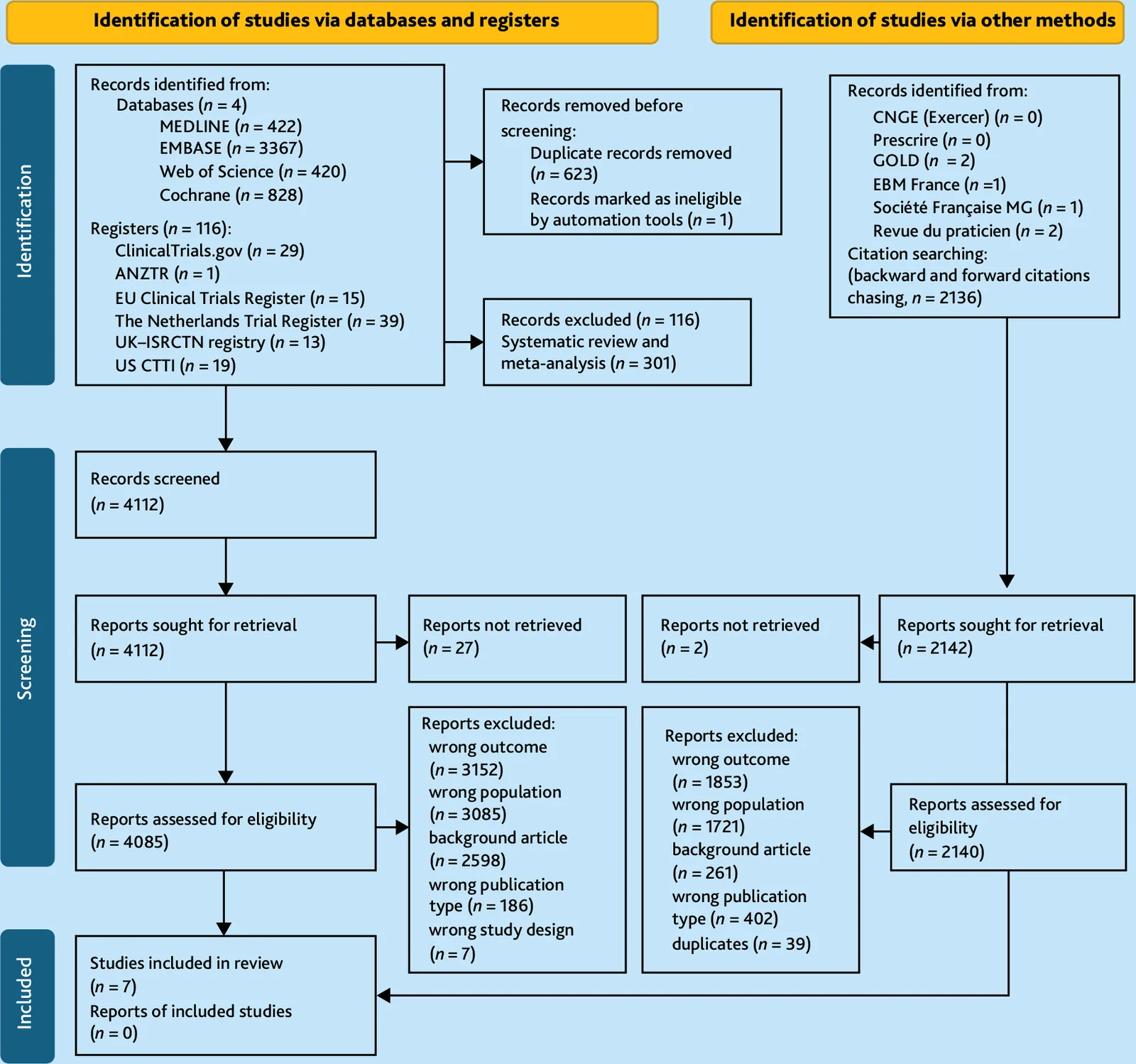
PRISMA 2020 flow chart diagram.^18^ Excluded reports can feature in multiple reasons. ANZTR = Australia New Zealand Trauma Registry. CNGE = Collège National des Généralistes Enseignants. ISRCTN = International Standard Randomised Controlled Trial Number. MG = de Médecine Générale.

The records selection is presented in Figure 1. Searches by keywords in databases and registers returned 4413 and 116 records, respectively, without duplicates. Records identified from other sources, including citation searching, returned 2142 records.

The four final search equations returned 422 records in MEDLINE, 3367 records in EMBASE, 420 records in Web of Science, and 828 records in CENTRAL. Records searched were mainly in English and rarely in French, German, or Spanish. At the end of the selection process, seven studies were included in the review:

- five quantitative studies related to CRP biomarkers as POCT (one RCT with 653 patients,^25^ two observational studies totalling 405 patients,^26,27^ and two before-and-after studies totalling 1449 patients^28,29^); and
- two RCTs related to PCT biomarkers as blood tests in laboratories with AECOPD subgroups including 21 and six patients.^30,31^

Two included observational studies were conducted by the same first author and were published only 1 year apart (Llor *et al* 2012 and Llor *et al* 2013).^27,28^ The first study examined a cohort in Spain, whereas the second involved a multinational cohort (Argentina, Denmark, Lithuania, Russia, Spain, and Sweden), though CRP POCT was mainly used in Sweden and Denmark. Therefore, these two studies did not address the same population.

The two PCT studies^30,31^ were included because at the abstract review stage it was interpreted that the primary outcome was the antibiotic prescribing rate with a PCT-guided approach. However, it turned out to be a secondary outcome. After discussion between reviewers, the authors decided to include them in the review for information purposes since no PCT study was used in the meta-analysis.

The RCT^25^ related to CRP biomarkers as POCT included in this study mentioned patient health outcomes as a secondary outcome. It indicated that less antibiotic use and fewer prescriptions from clinicians did not compromise patient-reported disease-specific quality of life.

One of the non-randomised studies, Strykowski *et al*,^29^ reported that underprescribing did not significantly increase. However, this may have been due to limitations in sample size. None of the other included studies addressed patient health outcomes.

A qualitative study^32^ based on the same English and Welsh population as the only RCT included that related to CRP biomarkers as POCT^25^ was excluded from this systematic review, as it did not provide quantitative data for the defined primary outcome. Interestingly, the study concluded that CRP POCT had high acceptability among primary care staff and patients, though commissioning arrangements and further simplification of the CRP POCT needed attention to facilitate implementation in routine practice.

Two studies reported cost analyses of CRP POCT (Sewell *et al* and Francis *et al*).^33,34^ Both were based on the RCT^25^ included in this review. These studies were excluded because the current systematic review aim did not encompass health economic outcomes. However, Sewell *et al,* ^33^ as the most recent and comprehensive cost analysis of CRP POCT, is considered in the Discussion section of this article.

CRP and PCT were the main biomarkers retrieved in the current review — CRP as a POCT and PCT in laboratories. Though leucocyte count appears in some records and considering a white blood count POCT device exists,^35^ no study assessed it as a biomarker to reduce antibiotic prescribing in AECOPD exacerbation in primary care. Likewise, neutrophil count exists as a POCT device^36^ and is mainly used for patients at risk of neutropenia following chemotherapy;^37^ however, no report mentioned its use in AECOPD exacerbation.

Eight records required discussion among the reviewers, and all were ultimately excluded, mainly owing to an incorrect publication type, an ineligible population, or an irrelevant outcome.^38–45^

### Not peer-reviewed literature

The grey literature was also explored but no records were included since by definition they were not approved through the official peer-review process. Five relevant theses were noticed but with a different population, with a different outcome, or were relatively old research works (from 2010) and without POCT analysis.

### Characteristics of the included studies

Table 1 reports the characteristics of the seven included quantitative studies.

**Table 1.** Characteristics of the included studies

Study lead author (year of publication)	Design	Patients, *n*	Location	Biomarker	Population	Primary outcome
Butler *et al* (2019)^25^	Open-label, randomised, controlled, multicentric, non-inferiority	653	England and Wales	CRP POCT	Patients aged ≥40 years with a COPD diagnosis registered in medical records and ≥1 Anthonisen criterion between 24 hours and 21 days	Antibiotic prescribing rate guided by CRP POCT versus standard care
Barber *et al* (2022)*^26^*	Quantitative, observational, retrospective	282	England	CRP POCT	Patients with a COPD diagnosis recorded in EMIS database used by 50% of GPs in England^58^ and ≥1 Anthonisen criterion	Antibiotic prescribing with CRP POCT versus standard care
Llor *et al* (2013)*^27^*	Cross-sectional, observational	123	Denmark and Sweden	CRP POCT	Patient with a spirometry diagnosis of COPD and ≥1 Anthonisen criterion	Antibiotic prescribing rate guided by CRP POCT versus standard care and analysis of predictors for antibiotic prescribing
Llor *et al* (2012)*^28^*	Prospective, non-randomised, controlled, before-and-after	783 (full-intervention group)	Spain	CRP POCT	Patients with a suspected LRTI (bronchitis, AECOPD, or pneumonia) by the doctor and based on symptoms and clinical signs (fever, coughing, dyspnoea, increase in sputum volume, and/or purulence of sputum)	Assess the effect of two interventions to reduce antibiotic prescribing in inferior RTI (training only versus training and CRP POCT)
Strykowski *et al* (2015)^29^	Prospective, non-randomised, controlled, before-and-after	666 (full-intervention group)	Spain	CRP POCT	Patients with a COPD diagnosis registered by a doctor and ≥1 Anthonisen criterion	Assess the effect of two interventions to reduce antibiotic prescribing in AECOPD (training only versus training and CRP POC)
Briel *et al* (2008)^30^	Open-label, randomised, controlled, multicentric, non-inferiority	458 (21 with AECOPD)	Switzerland	PCT in a laboratory	Patients with LRTI including a subgroup with AECOPD/asthma	Number of days, within the first 14 days after baseline, during which a patient’s daily activities were restricted by an RTI. Secondary outcome: antibiotic prescribing rate with a PCT-guided approach for LRTI
Burkhardt *et al* (2010)^31^	Randomised, controlled, non-inferiority	550 (6 with AECOPD)	Germany	PCT in a laboratory	Patients with LRTI including a subgroup with AECOPD/asthma	Number of days with significant health impairment due to acute RTI at 14 days. Secondary outcome: antibiotic prescribing rate with a PCT-guided approach for LRTI

AECOPD = acute exacerbation of chronic obstructive pulmonary disease. COPD = chronic obstructive pulmonary disease. CRP = C-reactive protein. LRTI = lower respiratory tract infection. PCT = procalcitonin. POCT = point-of-care test. RTI = respiratory tract infection.

### Risk of bias within studies

**Figure 2.**
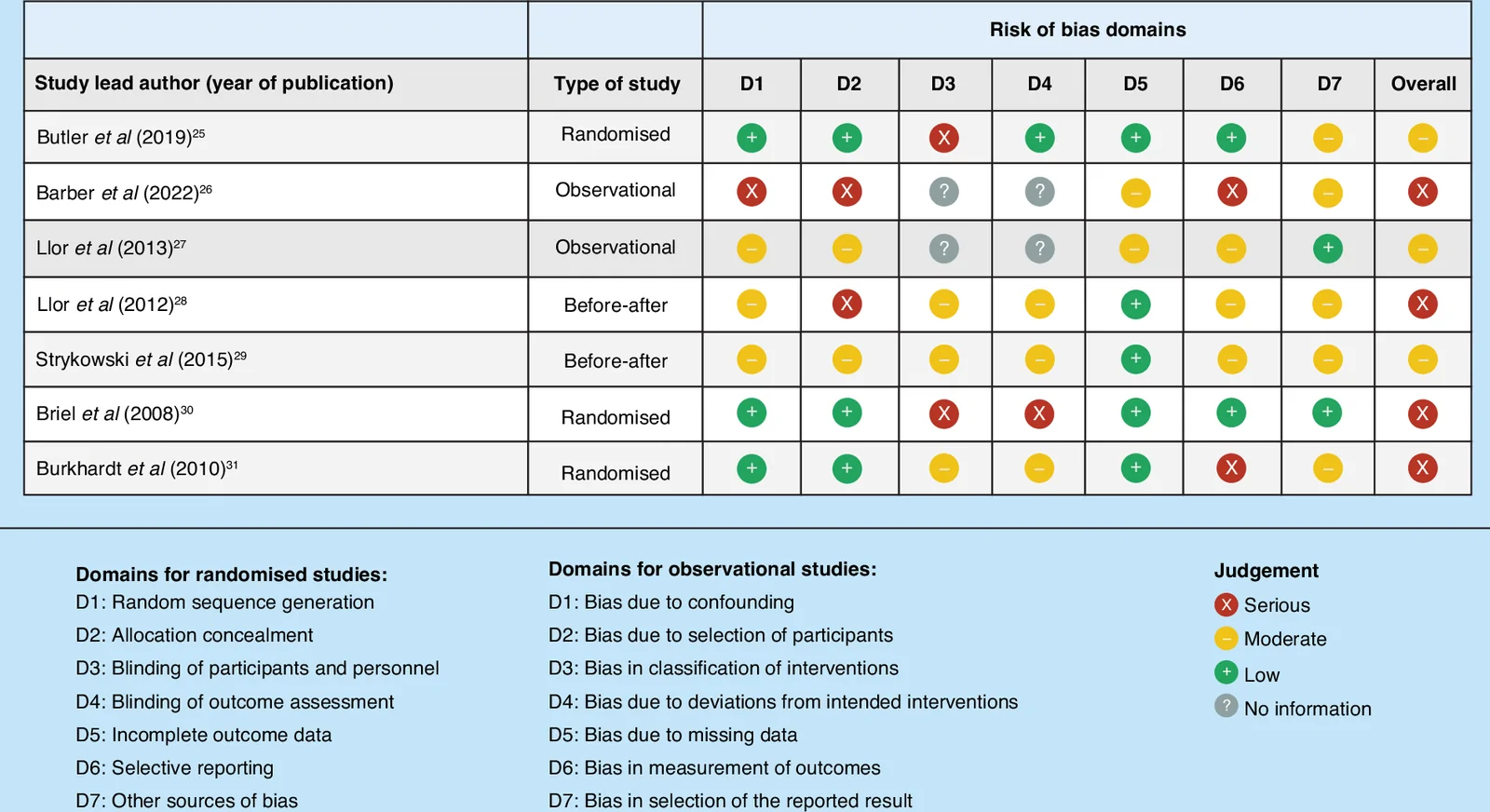
Risk of bias within studies. D = domain.

Figure 2 shows the biases within the studies. The RCT by Butler *et al* was well designed but had two noticeable biases: it was not blinded and the first author had a conflict of interest with Roche Molecular Systems (advisory board fees) and Roche Molecular Diagnostics (grant support) are a provider of POCT devices. However, another company, Abbott, loaned POCT devices for the study and had no role in the design, accrual, analysis, data interpretation, or manuscript preparation.^25^ This study had a good methodological quality, and a double-blind design may have proved difficult to implement in primary care with a POCT; therefore, a moderate overall bias was decided by both reviewers. Two non-randomised studies for CRP biomarkers had serious biases owing to confounding, selection of participants, or measurement of outcome.^26,28^

### Results of individual studies and level of evidence

All studies related to the CRP biomarker referred to POCT and found a statistically significant reduction in antibiotic prescribing rate for AECOPD (Table 2). The CRP cut-off for antibiotic prescribing from the best quality study was 40 mg/L, with no evidence of harm for patients.^25^ Other studies suggest either 50 mg/L,^26^ or a grey area between 20 mg/L and 100 mg/L where antibiotic prescribing should be discussed on a case-by-case basis (sputum purulence providing more weight to antibiotic prescribing).^27–29^ When CRP is ≥100 mg/L, antibiotics must be prescribed.^27–29^

**Table 2.** Main results of included studies

Study lead author (year of publication)	Main outcome: antibiotic prescription or use	Secondary outcomes: biomarker cut-off value and patient harm	Related instructions to biomarker cut-off value	Sensitivity, specificity, and AUC	Level of evidence (GRADE)
Butler *et al* (2019)*^25^*	CRP-guided group: 57.0% antibiotic use Usual care group: 77.4% antibiotic use 20.4% antibiotic reduction, adjusted OR 0.31 (95% CI = 0.20 to 0.47)	CRP <20 mg/L: antibiotics issued (32.8%) CRP ≥20–≤40 mg/L: antibiotics issued (84.2%) CRP >40 mg/L: antibiotics issued (94.7%) No evidence of patient harm by reducing antibiotic prescription. COPD-related health status, as measured by the Clinical COPD Questionnaire at 2 weeks after randomisation, was in favour of the COPD guided-group: adjusted mean difference in total score: –0.19 points (two-sided 90% CI = 0.33 to 0.05)	CRP >40 mg/L: antibiotic prescribing likely beneficial and should be prescribed CRP ≥20–≤40 mg/L: antibiotic prescribing beneficial if sputum purulence CRP <20 mg/L: antibiotic prescribing unlikely beneficial and should not be prescribed	CRP ≥40 mg/L: sensitivity 0.655; specificity 0.732; AUC 0.732 (95% CI = 0.614 to 0.851)^46^ CRP ≥40 mg/L and ≥1 AC (increased sputum purulence): AUC 0.842 (95% CI = 0.760 to 0.924)	Moderate
Barber *et al* (2022)*^26^*	CRP-guided group: 45.7% antibiotic use Usual care group: 70.1% antibiotic use 24.5% antibiotic reduction, OR 0.36 (95% CI = 0.21 to 0.61)^a^, *P*<0.001	CRP ≥50 mg/L when ≥2 AC (36.2%) and when <2 AC (8.8%); *P* = 0.008	CRP ≥50 mg/L: consider antibiotic prescribing	CRP ≥50 mg/L: sensitivity 0.94; specificity 0.72	Very low
Llor *et al* (2013)*^27^*	CRP-guided group: 54.3% antibiotic prescription Usual care group: 80.6% antibiotic prescription 26.3% antibiotic reduction, OR 0.34 (95% CI = 0.19 to 0.61), *P*<0.001	N/A	CRP ≤20 mg/L: do not prescribe antibiotics CRP >20–<100 mg/L: discuss antibiotic prescribing CRP ≥100 mg/L: antibiotics should be prescribed	N/A	Low
Llor *et al* (2012)^28^	CRP-guided post-intervention group: 72.9% antibiotic prescription Pre-intervention group: 82.3% antibiotic prescription 9.4% antibiotic reduction, OR 0.57 (95% CI = 0.41 to 0.80)	N/A	CRP ≤20 mg/L: do not prescribe antibiotics CRP >20–<100 mg/L: discuss antibiotic prescribing CRP ≥100 mg/L: antibiotics should be prescribed	N/A	Very low
Strykowski *et al* (2015)*^29^*	CRP-guided post-intervention group: 74.5% Pre-intervention group: 86.5% 12.0% antibiotic reduction, OR 0.46 (95% CI 0.31 to 0.68), *P*<0.001	Under-prescribing (in AECOPD with three AC) was not significant, so no patient harm: OR 0.25 (95% CI = 0.06 to 1.0 ), *P* = 0.075	CRP ≤20 mg/L: do not prescribe antibiotics CRP >20–<100 mg/L: discuss antibiotic prescribing CRP ≥100 mg/L: antibiotics should be prescribed	N/A	Low
Briel *et al (*2008)*^30^*	Considered as a secondary outcome PCT-guided group: 56% antibiotic use Usual care group: 92% antibiotic use No OR available for AECOPD subgroup	N/A	N/A	N/A	Low
Burkhardt *et al* (2010)*^31^*	Considered as a secondary outcome No OR available for AECOPD subgroup	N/A	N/A	N/A	Low

AC = Anthonisen criteria. AECOPD = acute exacerbation of chronic obstructive pulmonary disease. AUC = area under the curve. COPD = chronic obstructive pulmonary disease. CRP = C-reactive protein. GRADE = Grading of Recommendations Assessment, Development, and Evaluation. N/A = not applicable. OR = odds ratio. PCT = procalcitonin.

The two studies related to PCT biomarker (blood test in laboratories and not POCT) did not allow for a conclusion on the subgroup AECOPD because of an antibiotic prescribing rate evaluated as a secondary outcome and the absence of statistical analysis.^30,31^

### Quantitative study meta-analysis

**Figure 3.**
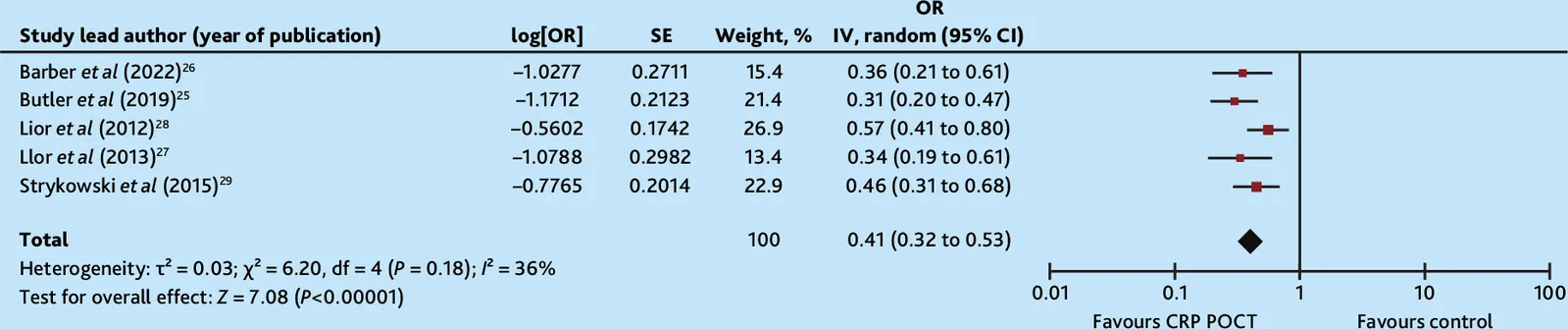
Meta-analysis of the five quantitative studies (CRP POCT). CRP = C-reactive protein. df = degrees of freedom. IV = inverse variance. OR = odds ratio. POCT = point-of-care test. SE = standard error.

Figure 3 shows the meta-analysis of the five quantitative studies for the CRP POCT, computed through the RevMan tool and totalling 2507 patients. It was in favour of an antibiotic prescribing reduction for AECOPD patients in primary care, with moderate homogeneity among studies: OR 0.41 (95% CI = 0.32 to 0.53); *I*² = 36%.

### Sensitivity analysis

The authors analysed sensitivity separately: non-randomised studies only,^26–29^ best quality studies (very low quality excluded),^25,27,29^ and studies with clear CRP cut-off values (CRP at 40 mg/L or 50 mg/L).^25,26^

The four non-RCT studies totalling 1854 patients were also in favour of the CRP POCT to reduce antibiotic prescribing with low heterogeneity: OR 0.46 (95% CI = 0.36 to 0.58); *I*² = 12%.^26–29^

The three best quality studies were also in favour of the CRP POCT to reduce antibiotic prescribing, without heterogeneity: OR 0.37 (95% CI = 0.29 to 0.48); *I*² = 0%.

The two studies with a clear CRP cut-off value at 40 mg/L and 50 mg/L also returned in favour of the CRP POCT to reduce antibiotic prescribing without heterogeneity: OR 0.33 (95% CI = 0.24 to 0.45); *I*² = 0%.

### Reporting biases

**Figure 4.**
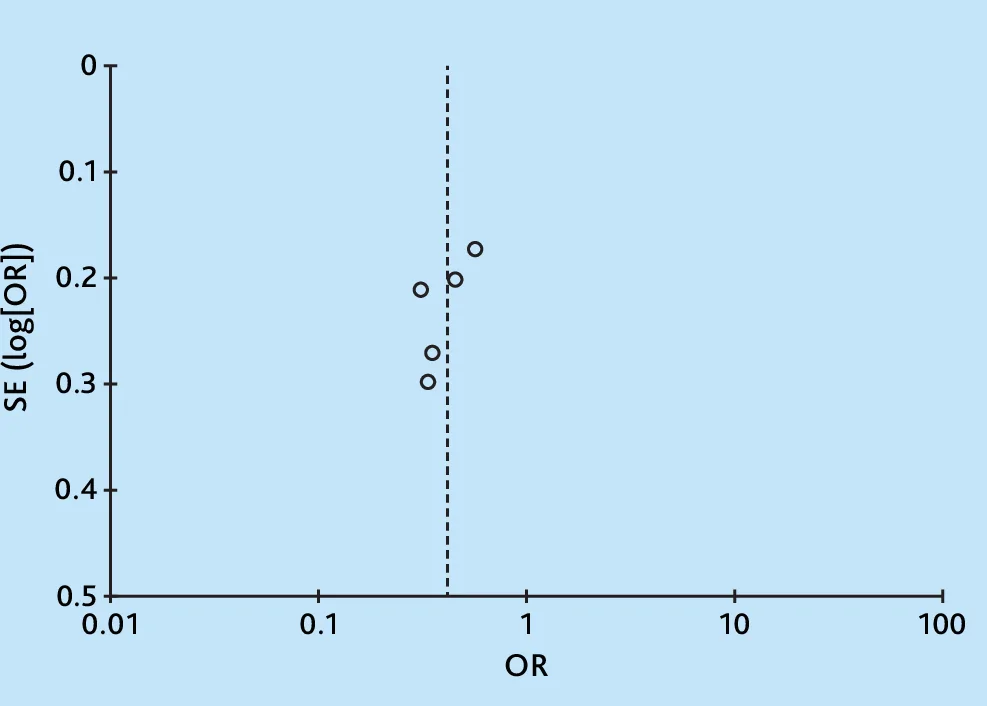
Funnel plot of the five quantitative studies (CRP POCT). CRP = C-reactive protein. OR = odds ratio. POCT = point-of-care testing. SE = standard error.

Regarding the overall publication bias, Figure 4 illustrates an acceptable risk with a well-balanced distribution of the results around the total OR.

### Certainty of evidence (GRADE)

In the CRP POCT studies, two non-randomised studies had a very low level of evidence due to significant biases,^26,28^ two had a low level of evidence,^27,29^ and the RCT had a moderate level of evidence.^25^ In the meta-analysis of the CRP biomarker, both reviewers evaluated the aggregated certainty of evidence as low (low to moderate if the sensitivity analysis with the three best quality studies is considered). Both randomised studies for PCT biomarkers had a low level of evidence (Table 2).^30,31^

## Discussion

### Summary

Of the seven quantitative studies: five related to CRP POCT, and two related to PCT as a blood test in laboratories. The CRP POCT studies included one RCT with 653 patients, and four non-randomised studies with a total of 1854 patients. The meta-analysis performed on one RCT and the four non-randomised studies for CRP POCT was in favour of an antibiotic prescribing reduction for patients with AECOPD in primary care.

Regarding secondary outcomes, it was difficult to aggregate different recommendations from the studies about CRP thresholds but in the single RCT, the cut-off value was set at 40 mg/L for antibiotic prescribing with an area under the curve (AUC) of 0.732.^25,46^ This threshold intended to support the clinical judgement and not replace it. If increased sputum purulence was observed, AUC for CRP POCT improved at 0.842,^11^ making it a quite efficient diagnosis tool without compromising patient safety.^25^

Only two of the five studies on CRP POCT, including the RCT, reported that the reduction in antibiotic prescribing did not result in patient harm or increased underprescribing as secondary outcomes.^25,29^

However, the certainty of evidence for the meta-analysis for CRP POCT was low (low to moderate if the sensitivity analysis with the three best quality is considered), mainly due to the lack of RCTs. The single RCT included was of good quality, yet open-labelled. The four remaining studies being non-randomised were at a higher risk of bias.

As the two studies investigating the effect of measuring PCT did not provide exploitable results for the AECOPD subgroup, and because the antibiotic prescribing rate was a secondary outcome, the evidence was very uncertain about the effect of measuring PCT for antibiotic prescribing reduction in primary care patients with AECOPD.

This systematic review and meta-analysis shows evidence to assert CRP POCT in significantly reducing antibiotic prescribing for AECOPD in primary care to support rather than replace clinical assessment. The evaluation of this intervention proves to be cost-effective in the UK.

### Strengths and limitations

To the authors’ knowledge, this is the first systematic review and meta-analysis addressing specifically an AECOPD population in primary care and assessing the effect of biomarkers to reduce unnecessary antibiotic prescribing as an adjunct to clinical examination. Data were gathered from broad sources: four databases, six registers, over 2000 citation searches, and six academic societies. Though some systematic reviews and meta-analyses were excluded from the search, citations and bibliographical references from these reviews were retrieved with the tool ‘citationchaser’, ensuring no relevant reports were missed.

In terms of limitations, the meta-analysis included one single RCT and four non-randomised studies. The lack of RCTs in the body of evidence limited the level of certainty. Combining RCTs and non-randomised studies with different designs and methods in a meta-analysis is not ideal, as mentioned in the *Cochrane Handbook for Systematic Reviews of Interventions*,^19^ though the results of the sensitivity analysis with non-randomised studies only remained close to the results of the five quantitative studies.

When the OR was not available within two non-randomised studies^26,28^ it was computed through the RevMan tool, but confounding factors may not have been taken into account leading to an unadjusted OR and a higher risk of bias (though these two studies were already considered the most biased in this review).

Regarding limitations related to included studies, the single RCT, the PACE trial, was open-labelled thus at risk of bias due to the lack of blinding. The main author had a conflict of interest with Roche Molecular Systems (advisory board fees) and Roche Molecular Diagnostics (grant support), a provider of POCT.^25^ For both reasons, this RCT study was downgraded from high to moderate level of evidence, despite the reputation of the journal in which it was published. The included non-randomised studies were of variable quality and did not always address patient harm as a secondary outcome, though antibiotic underprescribing may be an issue for patient safety in the case of serious bacterial infection. The level of evidence was assessed as low or very low for these non-randomised studies. Most included studies enrolled an AECOPD population based on physician-diagnosed COPD even though the official COPD definition is spirometry confirmed, albeit time consuming and not readily available in primary care settings.^47^ Even if the possibility of an alternate diagnosis, such as asthma, created a selection bias, this situation represents the real-word population being treated as patients with COPD in primary care.

### Comparison with existing literature

Sewell *et al*,^33^ in a cost analysis based on the PACE trial originally reported by Butler *et al,* ^25^ the only RCT included in this review, concluded that CRP POCT is a cost-effective intervention for safely reducing antibiotic consumption in patients with AECOPD. The cost of CRP POCT (£11.31 per test) was offset by savings in healthcare resource use related to COPD management.

The qualitative study, excluded from this systematic review, conducted on the PACE trial English and Welsh population concluded that CRP POCT had high acceptability among primary care staff and patients, though commissioning arrangements and further simplification of the CRP POCT need attention to facilitate implementation in routine practice.^32^

### Implications for research and practice

The authors suggest that more RCTs should be designed in a double-blind setting and without conflict of interest from POCT providers to confirm, on a bigger scale, that CRP POCT significantly reduces antibiotic prescribing for AECOPD. The academic society GOLD also suggested, in its latest 2024 report, that additional studies in other settings be conducted before a recommendation to generalise this approach.^2^ A CRP cut-off value for antibiotic prescribing should also be evaluated as a primary outcome to confirm a CRP cut-off value around 40 mg/L.

Evidence is very scarce about PCT measurement effect on antibiotic prescribing for AECOPD in primary care as no studies specifically focused on this population, particularly for PCT POCT. The cost of measuring PCT (about 10 times more expensive than CRP)^48^ also raises questions about its future viability in primary care at a time when healthcare spending is being carefully monitored.^49^

Other POCT biomarkers exist (mainly leucocytes and neutrophils),^35,36^ but, to the authors’ knowledge, no study has assessed them for reducing antibiotic prescribing for AECOPD in primary care. Some studies in secondary care suggest combining CRP with other biomarkers might enhance performance diagnosis in case of bacterial AECOPD.^50–52^ A dual or triple biomarker (CRP/neutrophils or CRP/leucocytes/neutrophils) might then identify AECOPD requiring antibiotic prescribing more accurately.

Besides antibiotics, GPs often prescribe oral glucocorticoids to treat AECOPD in primary care and this treatment may be guided by an eosinophil POCT. In a 2024 multicentre, double-blind, randomised controlled trial in 14 primary care settings in the UK with 308 patients, treating eosinophilic AECOPD only with corticosteroids did not make a significant difference to re-treatment, hospitalisations, or deaths compared with treating all AECOPD with corticosteroids.^53^ Thus, such biomarkers could identify COPD exacerbation phenotype to guide not only antibiotic use, but also oral glucocorticoid therapy in primary care. This could be a promising path to a more personalised treatment plan, respectful to the ecosystem and alleviating unnecessary costs to primary healthcare systems.

Regarding the practical feasibility, POCT devices in primary care are well implemented and regularly used in many European countries (Denmark, Netherlands, Norway, and Switzerland).^54,55^ However, in France, medical biology outsourcing in GP practices is poorly developed and has no legal basis since blood tests must be executed and validated in laboratories only.^56^ Reluctance among some healthcare professionals to change to outsourcing biology has been reported.^57^

## Data

All data are available on request from the first author (mathieu.frenois@gmail.com).
